# Antagonism of Bradykinin B2 Receptor Prevents Inflammatory Responses in Human Endothelial Cells by Quenching the NF-kB Pathway Activation

**DOI:** 10.1371/journal.pone.0084358

**Published:** 2014-01-02

**Authors:** Erika Terzuoli, Stefania Meini, Paola Cucchi, Claudio Catalani, Cecilia Cialdai, Carlo Alberto Maggi, Antonio Giachetti, Marina Ziche, Sandra Donnini

**Affiliations:** 1 Department of Life Sciences, University of Siena, Siena, Italy¸; 2 Pharmacology Department, Menarini Ricerche S.p.A, Florence, Italy; University of Bristol, United Kingdom

## Abstract

**Background:**

Bradykinin (BK) induces angiogenesis by promoting vessel permeability, growth and remodeling. This study aimed to demonstrate that the B2R antagonist, fasitibant, inhibits the BK pro-angiogenic effects.

**Methodology:**

We assesed the ability of fasibitant to antagonize the BK stimulation of cultured human cells (HUVEC) and circulating pro-angiogenic cells (PACs), in producing cell permeability (paracellular flux), migration and pseocapillary formation. The latter parameter was studied in vitro (matrigel assay) and in vivo in mice (matrigel plug) and in rat model of experimental osteoarthritis (OA). We also evaluated NF-κB activation in cultured cells by measuring its nuclear translocation and its downstream effectors such as the proangiogenic ciclooxygenase-2 (COX-2), prostaglandin E-2 and vascular endothelial growth factor (VEGF).

**Principal findings:**

HUVEC, exposed to BK (1–10 µM), showed increased permeability, disassembly of adherens and tight-junction, increased cell migration, and pseudocapillaries formation. We observed a significant increase of vessel density in the matrigel assay in mice and in rats OA model. Importantly, B2R stimulation elicited, both in HUVEC and PACs, NF-κB activation, leading to COX-2 overexpression, enhanced prostaglandin E-2 production. and VEGF output. The BK/NF-κB axis, and the ensuing amplification of inflammatory/angiogenic responses were fully prevented by fasitibant as well as by IKK VII, an NF-κB. Inhibitor.

**Conclusion:**

This work illustrates the role of the endothelium in the inflammation provoked by the BK/NF-κB axis. It also demonstates that B2R blockade by the antaogonist fasibitant, abolishes both the initial stimulus and its amplification, strongly attenuating the propagation of inflammation.

## Introduction

The inflammation elicited by bradykinin (BK) through the B1 and B2 receptors (B1R, B2R) recapitules the cardinal signs of an inflammatory response as it induces: vascular permeability, hyperthermia, oedema, pain and neo-vessel formation (angiogenesis) [1]–[7]. More recently, BK has been described to be involved in the pathogenesis of degenerative joint diseases, such as the knee osteoarthritis [8]–[10]. During the osteoarthritis process, chronic inflammation promotes the imbalance of metabolic and degradative signals. BK, through the B2R, contributes to the chronic inflammatory response in the knee osteoarhritis, activating different cells, including synovial cells or chondrocytes, and inducing the release of pro-inflammatory cytokines, as well as the products of ciclooxygenase (COX) and lipooxygenase (LOX) [10], [11]. Several peptide and non-peptide B2R antagonists have been synthesised [12], [13]. Icatibant, a peptide compound, is one of the first B2R antagonists synthesised, now approved for the therapy of hereditary angio-oedema attacks [7], [14]. Recently, the non-peptide B2R antagonist fasitibant (formerly MEN16132) showed a remarkably high affinity and antagonist potency toward B2R in different species, including humans [15]–[18]. In preclinical models of inflammation and pain, including osteoarthritis, fasitibant was effective and long lasting in blocking both exogenous and endogenous BK [19]–[22]. The compound is now undergoing a phase II clinical study in knee osteoarthritis patients (ClinicalTrials.gov: NCT01091116).

Angiogenesis plays a pivotal role in the advancement of inflammatory diseases progression, including osteoarthritis, as a source of inflammatory cells, cytokine and protease activity [23].

Vascular growth both in the synovium and at the osteochondral junction have been associated with osteoarthritis, which is characterized by synovitis and progressive cartilage degeneration, thus novel therapies capable to limit angiogenesis, besides inflammation and pain, might be a desirable target [24], [25]. Notably, in the progression of osteoarthritis, the benefits of agents that suppress neovascularization has been very impressive, providing a solid rationale for pursuing anti-angiogenesis strategies in patients affected by chronic inflammatory diseases [26]. Of relevance to this study are recent reports describing the role of the kallikrein/bradykinin system, through the B2R in the recruitment of circulating pro-angiogenic cells, a process which leads to tissue vascularization [27].

BK is known to induce angiogenesis by activating endothelial cells, and promoting vessel permeability, growth and remodeling [28], [1], [4], [5]. The present study aimed to demonstrate that the B2R antagonist, fasitibant, inhibits the BK pro-angiogenic effects, both in *in vitro* and *in vivo* studies. Moreover, we provide evidence that, in endothelial cells and in circulating proangiogenic cells (PACs), BK activates the pro-inflammatory NF-κB transcriptional factor, which, in turn, promotes the overexpression of a wide array of inflammatory genes (e.g. interleukins, chemokines, COX-2, MMPs). As a measure of the functional NF-κB activation, we assessed the COX-2/prostaglandin E-2 (PGE-2) pathway, because of their established role as pro-inflammatory and pro-angiogenic signals. We demonstrate that fasitibant abolished pro-angiogenic effects by suppressing the B2R-dependent BK-induced NF-κB transcription factor activation.

## Results

### HUVEC Express B2 but not B1 Receptors

**Figure 1:**
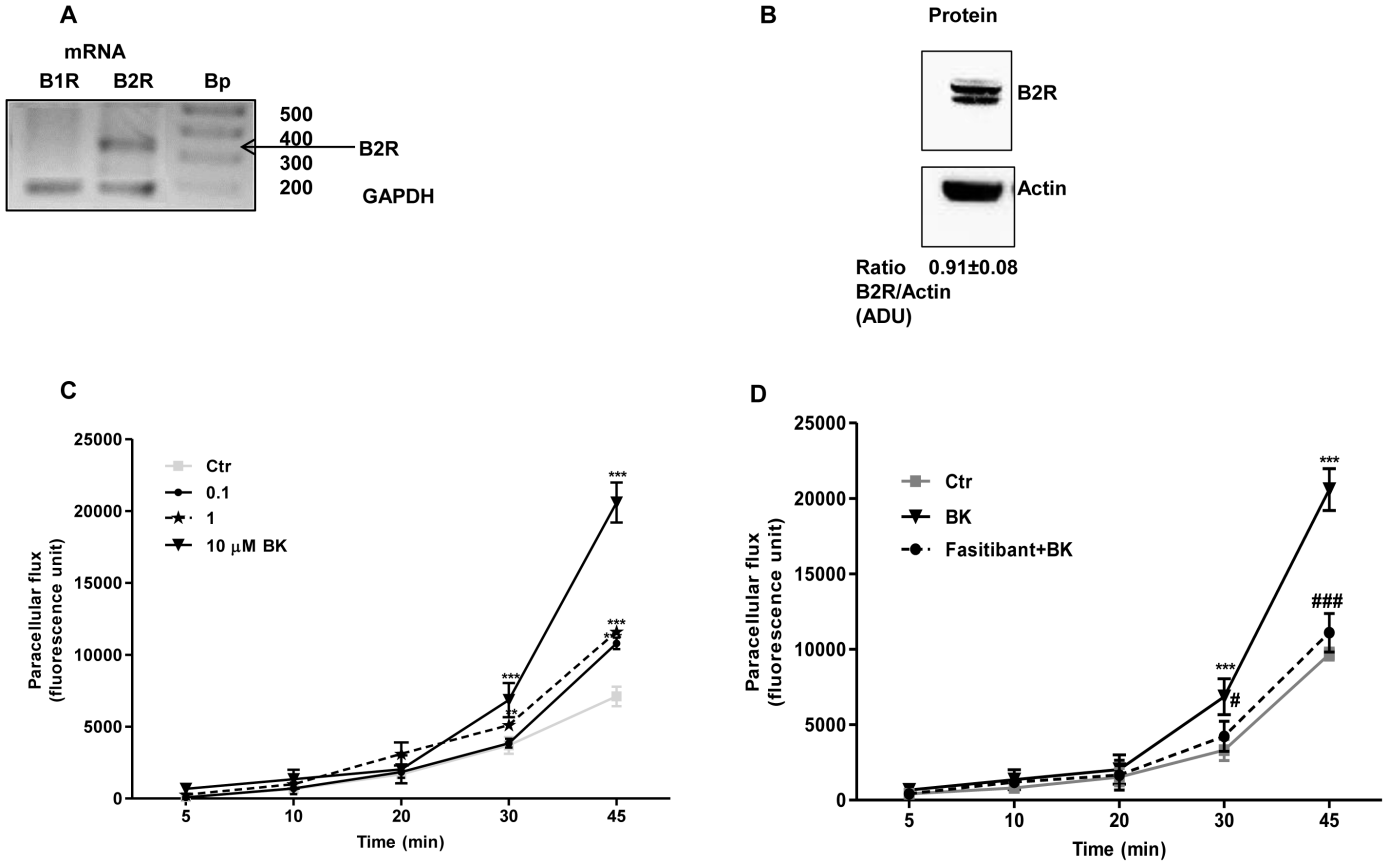
B2R stimulation promotes changes of cell permeability. (A) mRNA expression for B1 and B2 receptors, (B2R mRNA is approximately 339 pb) and (B) western blot analysis of B2 receptor in HUVEC. (Experiments are run three time; n = 3). (C) Permeability in HUVEC monolayer was detected as passage of fluorescence-coniugated FITC-Dextran from upper to lower compartments (Numbers represent mean ± SEM of three experiments run in triplicate; n = 3); ***p<0.001, **p<0.01, *p<0.05 compared to untreated cells (D). Fasitibant (1 µM), prevents the enhanced permeability, n = 3; ***p<0.001 compared to untreated cells #p<0.05, ###p<0.001 to BK-treated cells.

In order to evaluate the anti-angiogenic activity exerted by fasitibant on cultured human umbelical venular endothelial cells, HUVEC, first, we assessed the presence of the BK receptors, B1 and B2, by measuring their respective mRNA and protein expression (Fig. 1A and B). The B2R was revealed in terms of messenger (Fig. 1A) and protein, and it was not modulated by the presence of BK (Fig. 1B and Fig. S1). The B1R was undetectable in HUVEC (Fig. 1A), either in basal condition (0.1% FCS) after BK or 10% FCS stimulation (Fig. S1).

### Fasitibant Prevents Changes in Permeability, and Adherens and Tight-junction Signals Induced by BK

Endothelial permeability, a common histopatological marker of inflammation, is also a typical response to several angiogenic factors [29]. In a model of *in vitro* endothelial permeability, BK produced a significant increase of paracellular flux of fluorescent-conjugated dextran, which was time- and concentration-dependent (Fig. 1C). Cotreatment of HUVEC with BK (10 µM) and fasitibant (1 µM), abolished the BK-induced paracellular flux increase, restoring the flux to control level (Fig. 1D). A lower fasitibant concentration (0.1 µM) produced a non-significant inhibition (data not shown).

**Figure 2:**
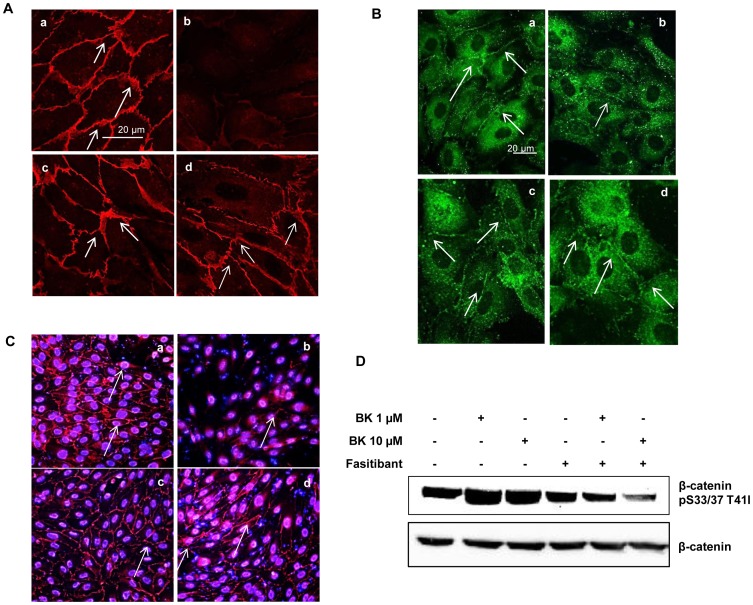
BK-induced changes of endothelial junctions signals are blocked by fasitibant in HUVEC. (A) Confocal analysis of VEC expression (white arrowheads) in 0.1% FBS (a), BK (1 µM) (b), fasitibant (1 µM) (c), fasitibant+BK (d). (B–C) ZO-1 (60 X) and VEC (20 X) expression (white arrowheads), evaluated by immunofluorescence analysis, in 0.1% FBS (a), BK (1 µM) (b), fasitibant (1 µM) (c), fasitibant+BK (d). Bar = 20 µM. (D) Cytoplamic β-catenin phosphorylation, (western blot), in cells treated with BK (1 or 10 µM) with/without fasitibant (1 µM). Gels representative of three experiments; n = 3.

In a condition of *in vitro* confluence, cells regulate permeability through the expression of cell-type-specific transmembrane adhesion proteins, such as vascular endothelial-cadherin (VEC), at adherens junctions, and zonula occludens-1 (ZO-1), at tight junctions. Consistent with its permeability effects, BK drastically reduced the typical pattern of fluorescence localization of either VEC (Fig. 2A, panel b vs. panel a and Fig. S2 A) or ZO-1 (Fig. 2B, panel b vs. panel a and Fig. S2 A) at the cell-cell contacts, as shown by the white arrows in panels a vs. b in both Figs. 2A and B. Fasitibant restored the cytoskeletal organization of both molecules (Fig. 2A, panel d, and Fig. 2B, panel d). Further analysis of VEC expression by immunofluorescence at a lower magnification (20X) clearly depicts the dramatic reduction of adherens junction in response to BK (Fig. 2C, panel b vs. a) contrasting with that of fasitibant treatment (Fig. 2C, panel d vs. b).

It is known that VEC expression decreases β-catenin phosphorylation/translocation into the nucleus [30]. Consistent with the observed disassembly of VEC, BK (1 or 10 µM) increased cytoplamic β-catenin phosphorylation, an effect fully reversed by fasitibant (Fig. 2D, compare lane 5 and 6 vs. 2 and 3, Fig. S2 B for quantification).

These observations clearly indicate that fasitibant prevents BK-induced permeability by counteracting the disassembly of adherens and tight junction.

### Fasitibant Inhibits BK-induced Angiogenesis

**Figure 3:**
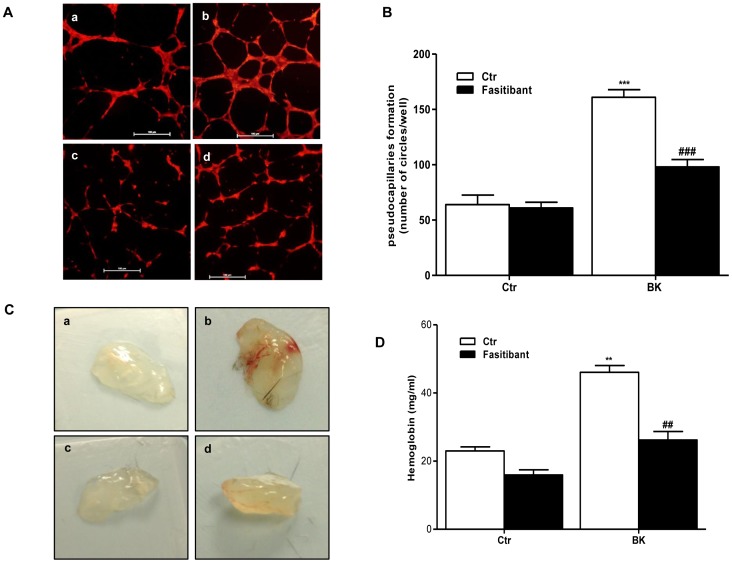
B2R blockade reduces BK-induced angiogenesis. (A) Representative pictures of pseudocapillaries formation in Matrigel from HUVEC in 0.1% FBS (a), exposed to BK (1 µM) (b), to fasitibant (1 µM) (c), to fasitibant+BK (d), observed 12 hrs after cell seeding. (B). Quantification of pseudocapillaries obtained by counting numbers of complete circles/well; Numbers represent mean ± SEM of three experiments run in triplicate. (C) BK induces vascularization in subcutaneously-injected Matrigel implants in mice. panel a: none, b: BK, c: fasitibant and d: fasitibant+BK. (D) Quantitative analysis of hemoglobin/angiogenesis in implants. For each condition (n = 6), the means ± SD are shown. **p<0.01, compared to untreated cells; ##P<0.01 to BK-treated cells.

BK promotes angiogenesis by directly stimulating endothelial cell (EC) growth and migration [4]. In HUVEC, BK induced both a concentration-dependent cell proliferation (measured by the incorporation of BrdU) and migration (measured by the wound healing assay), with a maximal effect at 1 µM (data not shown). Fasitibant (1 µM) co-incubated with BK (1 µM), suppressed its effects (Fig. S3 A and B, Fig. S3 C for wound healing assay quantification). Further, the anti-angiogenic effect of fasitibant was evident also in a 3D differentiation model. When plated in a thin layer of matrigel and stimulated with BK, HUVEC organized in a network of pseudocapillary tubes that invaded the gel (Fig. 3A, panel b vs. a). Fasitibant co-treatment reduced the number of pseudocapillary in terms of completed circles (Fig. 3A, panel d vs. b and Fig. 3B for quantification). Similarly, BK promoted pseudocapillary sprouting in a microcarrier-beads-HUVEC model (Fig. S4 A and B for quantification). Fasitibant co-treatment abolished the cellular sprouting, affecting not only the number and length of BK-induced sproutings, (Fig. S4 A, panel d vs. b, and B for quantification), but reducing (p<0.001) capillary diameter (Fig. S4 C, panels b vs. a, and D for quantification).

To further test the anti-angiogenic activity of the B2R antagonist, we used the Matrigel implant assay. Matrigel plugs, both with BK alone or in presence of fasitibant, were injected subcutaneously in mice and harvested after 10 days. Implants containing BK showed several branched structures throughout the implant (Fig. 3C, panel b). Conversely, in implants in which BK and fasitibant were concomitantly administered, angiogenesis was markedly reduced (Fig. 3C panel d vs. b). Quantitative analysis of viable/functioning vessels by hemoglobin determination revealed that BK (10 µM) produced a 2.3-fold increase in blood content compared with control (Fig. 3D), whereas administration of fasitibant (1 µM) reduced vessel density by 50% (Fig. 3D). All together the results demonstrate that the B2R antagonist impairs BK-dependent endothelial cell activation and angiogenesis.

### Angiogenesis and Progenitor Hematopoietic Cells in Experimental Osteoarthritis in Rats: Effect of B2R Antagonism

**Figure 4:**
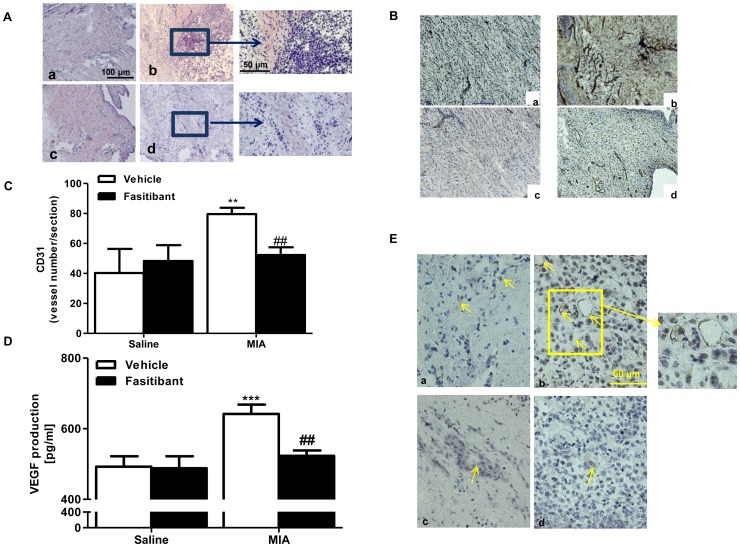
Effect of fasitibant on angiogenesis and circulating proangiogenic cells in experimental osteoarthritis in rats. (A) Representative images of hematoxylin/eosin staining (20 X) in synovial tissue from rats after intra articular injection of: saline (a), MIA (b), fasitibant with saline (c) or MIA with fasitibant, as described in Materials and Methods. Bar = 100 µM. Inset at higher magnification (40 X). Bar = 50 µM. (B–C) Representative images and quantification of CD-31 staining (40 X) in rats as described in A; Bar = 50 µM; quantification of CD-31 was performed counting 10 random field/section for slides; each slide has four sections. Data represent vessels counted for section in synovial tissue **P<0.01 compared to saline; ##P<0.01 compared to MIA. (D) ELISA immunoassay for VEGF in synovial fluids of rats treated as described in A. ***P<0.001 compared to saline; ##P<0.01 to MIA. (E) Representative images of CD-133 staining in rats as described in A; Bar = 50 µM. Inset at higher magnification (40 X).

We assessed the relevance of fasitibant on angiogenic process in a model of experimental (intra-articular administration of monosodium iodoacetate, MIA) osteoarthritis in rats. In this model, we observed a marked infiltration of inflammatory cells with dearrengment of synovial tissue structure, as evaluated by hematoxylin and eosin staining, which was reduced by simultaneus fasitibant administration (Fig. 4A). Moreover, MIA administrationin produced a significant increase of vessel density, evidenced by CD31 staining (Fig. 4B panel b vs. panel a, and Fig. 4C, p<0.01). The co-treatment with fasitibant significantly reduced the number of vessels (Fig. 4B, panel d, and Fig. 4C), demonstrating anti-angiogenic properties. Next, measurements of VEGF levels in synovial fluids (Fig. 4D), showed significantly higher levels in MIA treated rats (640±26 pg/ml) compared to control (490±29 pg/ml, p<0.001, Fig. 4D). Fasitibant, significantly reduced VEGF levels (520±15 pg/ml, P<0.01, Fig. 4D).

A number of reports support the notion that circulating proangiogenic cells (PACs) foster angiogenesis in selected diseases, including osteoarthritis [26]. Indeed we found a significant enrichment of progenitor cells, evidenced by CD133 staining and localised around the vessels, in the MIA treated rats (Fig. 4E panel b vs. panel a), that was reduced by concomitant administration of fasitibant (Fig. 4E, panel d).

### BK Activates the NF-κB-signaling Pathway Producing a Persistent Activation of Human Endothelial Cells

**Figure 5:**
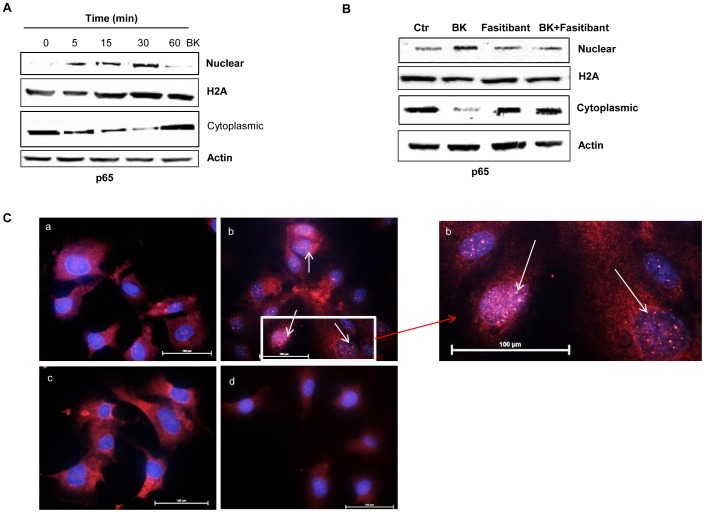
BK stimulates translocation/activation of NF-κB in HUVEC. (A) NF-κB translocation following BK (1 µM) exposure for the indicated times, (B) or following exposure to BK (1 µM, 30 min) in presence/absence of fasitibant (1 µM). Gel are representative of three experiments. (C) Immunofluorescence analysis (40 X) of NF-κB translocation in HUVEC in 0.1% FBS (a), BK (1 µM) (b), fasitibant (1 µM,) (c), fasitibant+BK (d). Inset at higher magnification. Bar = 100 µM.

Next, to learn how the acute and short lived inflammatory responses elicited by BK in the human endothelium would lead to a chronic endothelial activation, a condition sufficient to promote the degenerative pathologies of the joints [31], we studied the transcription factor NF-κB, a master regulator for the inflammatory cascade. We therefore examined NF-κB translocation from the cytoplasm into the nucleus, a fundamental index of NF-κB gene transcriptional activation, using western blot analysis for anti-p65 NF-κB antibody. BK stimulated NF-κB translocation rapidly, as its presence in the nucleus was detectable as early as 5 min, persisting up to 30 min following BK (Fig. 5A, Fig. S5 A for quantification). Importantly, the B2R antagonist prevented the nuclear translocation of the transcription factor (15 min), and the ensuing activation of the inflammatory pathways, evaluated by western blot and immunofluorescence (Fig. 5B and C, Fig. S5 B for quantification).

**Figure 6:**
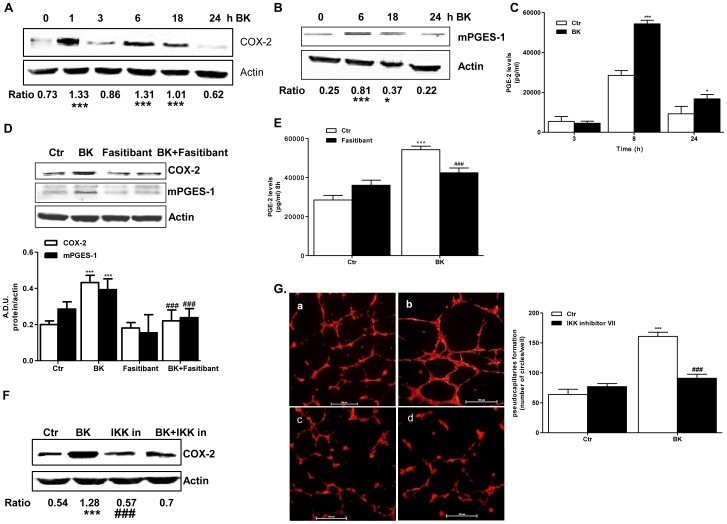
Fasitibant suppresses BK-induced COX-2 signaling. (A–B) COX-2 and mPGES-1 expression (western blot) in HUVEC treated with BK (1 µM) for the indicated times. Gels are representative of three experiments. The ratio between COX-2 or mPGES-1 over actin is reported. *p<0.05, ***p<0.001, compared to untreated cells. (C) PGE-2 release in the conditioned medium of HUVEC treated with BK (1 µM) for the indicated times. All PGE-2 release experiments in this paper were performed in archidonic acid pre-treated cells. Numbers represent mean ± SEM of three experiments. *p<0.05, ***p<0.001, compared to untreated cells; (D) COX-2 and mPGES-1 expression in HUVEC treated with BK (1 µM, 6 hrs) with/without fasitibant (1 µM). Gels are representative of three experiments. Graphs represent the optical densities related to the ratio between COX-2 or mPGES-1 over actin. A.D.U. (arbitrary density unit), numbers represent mean ± SD of three experiments ***p<0.001, compared to untreated cells; ###P<0.001 to BK-treated cells. (E) PGE-2 release from HUVEC treated with BK (1 µM) in presence/absence of fasitibant (1 µM), for 8 hrs; Numbers represent mean ± SEM of three experiments. ***p<0.001, compared to untreated cells; ###P<0.001 to BK-treated cells; (F) COX-2 expression (western blot) in HUVEC pretreated for 30 min with IKK inhibitor VII (0.2 µM), and treated with BK (1 µM, 6 hrs). Gel is representative of three experiments. The ratio between COX-2 over actin is reported. ***p<0.001 compared to untreated cells; ###P<0.001 to BK-treated cells. (G) Representative pictures and quantification of pseudocapillary formation in Matrigel by HUVEC exposed to 0.1% FBS (panel a), BK (1 µM, panel b), IKK inhibitor VII (0.2 µM, panel c) with or without BK (1 µM, panel d), observed at 12 hrs after cell seeding. Quantification was obtained as above, ***p<0.001, compared to untreated cells; ###P<0.001 to BK-treated cells. Numbers represent mean ± SEM of three experiments.

NF-κB activation entails overexpression of a number of genes and their pro-inflammatory products (e.g. cytokines, MMPs, COX-2) which promote a chronic type of inflammation and further stimulate the endothelium to form neo-vessels. In this work on human endothelium we focused on inducible enzymes of the arachidonic acid pathway, i.e. COX-2 and mPGES-1, because their product, PGE-2, is widely recognized as an important factor in fostering pathological angiogenesis [32]. We observed that BK induced a consistent biphasic over-expression of COX-2 (at 1 and at 6 hrs following BK exposure) and of mPGES-1 (Fig. 6A and B). The enhanced expression of these synthetic enzymes yielded a nearly twofold increase of PGE-2 production (Fig. 6C). Simultaneous incubation of HUVEC with BK and fasitibant reduced the overexpression of both COX-2 and mPGES-1 (Fig. 6D), and significantly attenuated the PGE-2 output (Fig. 6E; p<0.001). The level of PGE-2 production in the endothelium greatly influenced its functions, such as migration and pseudocapillary formation, which were enhanced by BK exposure, and conversely inhibited by fasibitant. Similarly, the IKK inhibitor VII (0.2 µM), nearly suppressed the BK-induced COX-2 overexpression (Fig. 6F) and affected also the formation pseudocapillaries in matrigel (Fig. 6G, panel d versus panel b).

**Figure 7:**
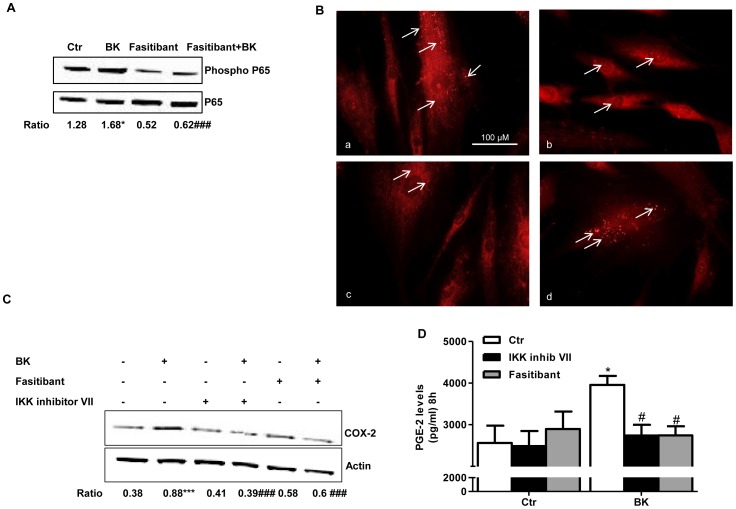
BK stimulates translocation/phosphorylation of NF-κB in circulating proangiogenic cells. (A) p65 (NF-κB) phosphorylation following exposure to BK (1 µM, 15 min) in presence/absence of fasitibant (0.1 µM). Gel are representative of three experiments. The ratio between p-p65 over p65 is reported. *p<0.05 compared to untreated cells; ###P<0.001 to BK-treated cells. (B) Immunofluorescence (40 X) of NF-κB translocation in PACs in 0.1% FBS (a), BK (1 µM) (b), fasitibant (0.1 µM,) (c), fasitibant+BK (d). Bar = 100 µM. (C) COX-2 expression in human hematopoietic progenitor cells pretreated for 30 min with IKK inhibitor VII (0.2 µM) or with fasitibant (0.1 µM), and treated with BK (1 µM, 6 hrs). Gel is representative of three experiments. The ratio between COX-2 over actin is reported. ***p<0.001 compared to untreated cells; ###P<0.001 to BK-treated cells. (D) PGE-2 release from PACs treated with BK (1 µM) in presence/absence of fasitibant (0.1 µM) or IKK inhibitor VII (0.2 µM), for 8 hrs. Numbers represent mean ± SEM of three experiments. *p<0.01, compared to untreated cells; #P<0.01 to BK-treated cells.

Interestingly, we found similar results in PACs (Fig. 7). PACs expressed B2R (Fig. S6), and BK induced NF-kB activation, evaluated as nuclear translocation and phosphorylation of p65, COX-2 expression and PGE-2 production (Fig. 7A–D), which were reduced by treatment with fasitibant (0.1 µM), or IKK inhibitor VII (0.2 µM).

All together these results demonstrate that BK, the initial stimulus for the activation of the NF-κB pathway, has important consequences for the human endothelium as it amplifies the inflammatory response by involving enhanced expression of genes and their pro-inflammatory products. These results also clerly show that blockade of the initial stimulus through kinin B2 receptor blockade by fasitibant abolishes the BK/NF-κB axis and prevents the progression of inflammation.

## Discussion

This work delineates the profile of a novel selective B2R antagonist, fasitibant, in blocking the BK-induced activation of vascular endothelium. Fasitibant binds to the human B2R exhibiting a sub-nanomolar affinity, similar to that of the peptidic antagonist icatibant (pK_i_ 10.1–10.5), whereas in virtue of its dissociation-rate remarkably slower [33] fasitibant displays a significantly higher functional potency in blocking BK induced responses (pK_B_ values 10.3 and 8.5 for fasitibant and icatibant, respectively) [15]. Moreover, fasitibant, in an *in vivo* model of osteoarthritis, has been shown to reduce oedema, to possess a long lasting pain-relieving activity, and to lower the output of BK and prostanoids in the joint [21].

Because oedema formation is associated with an increased leakage from the blood vessel of the joint, its is likely that the compound opposes the effect of BK on the endothelial cells lining these vessels. Indeed, this work demonstrates that fasitibant antagonizes the activation of endothelial cells induced by BK, abolishing the massive increase of microvascular permeability. Similarly, fasitibant preventes the BK-induced changes of endothelial signals indicative of faulty membrane, i.e. the VE-cadherin (VEC) and zonula occludens (ZO)-1 proteins and β-catenin phosphorylation. Thus, blockade of the B2R preserves the charateristic barrier function of the endothelium, by preventing the disorganization of the adherens and tight junctions *in face of* injurious stimuli, and exerts a tight control on the efflux rate.

A major finding concerns the BK ability to activate the trancription factor NF-κB, as evidenced by its translocation toward the cell nucleus, an event which occurs within minutes from BK addition to endothelial cells. NF-κB, a pivotal regulator of inflammation [34], [35], in its transcriptional role, subserves a wide repertoire of inflammatory genes (cytokines, growth factors, enzymes). The delayed overexpression of COX-2 and overproduction of PGE-2, observed in HUVEC challenged with BK, represent a clear functional evidence of the involvement of the NF-kB in gene regulation. Thus, it appears that BK functions as the initial trigger for a robust chronic inflammatory response sustained by a wide variety of inflammatory genes and mediators.

Notably, fasibitant application to endothelial cells aborted the NF-κB nuclear translocation, therefore inhibiting the functional sequelae of its activation. Similarly, IKK VII, an inhibitor of NF-κB, prevented endothelial functions related to its active state.

Thus, these results delineate distinct, but overlapping phases of the inflammatory response to BK in the endothelium. An acute one, possibly involving fast signals, such as VEC and ZO-1 disassembling, with phoshorylation of beta catenin, and NF-κB translocation, followed by a chronic phase, involving gene transcription and protein synthesis initiated by NF-κB transcriptional activity. This amplification mechanism of local and short-lived signals, such as that of BK, exhaustively reviewed by Pober and Sessa [31], is crucial for the evolution of inflammation. Antagonism of B2R, completely suppresses this progression.

The endothelium, activated through B2 receptor stimulation and by the NF-κB cascade products, exibits the typical angiogenic phenotype, as shown by the abundant sprouting of new capillaries observed in this work in various *in vitro* and *in vivo* assays. This effect was preceeded by growth and enhanced motility of endothelial cells. Particularly significant were the results *in vivo* on the matrigel plug, which illustrate the invasion of blood capillaries of the plug under BK challenge and their suppression provided by fasibitant.

We also reproduced these effects in the in vivo experimental model of OA [21]. Indeed, treatment with MIA induced a marked inflammatory response in the rat joint, characterized by enhanced microvessel density and enrichment of circulating proangiogenic cells (PACs). In vitro, in cultured PACs, again we observed the BK mediated amplification of the inflammatory response, as shown by NF-kB activation and COX-2-PGE-2 pathway activation. All these effects were blocked by fasitibant aplication. Corroborating these findings, a recent report shows the stringent requirement for NF-κB activation in the process of arteriogenesis [36].

In conclusion this work illustrates that blockade of the B2 receptor by a selective antagonist, prevents the acute inflammatory responses of the vascular endothelium, and aborts the ensuing amplification through the NF-κB pathway, and its propagation associated to pathological angiogenesis.

## Methods

### Reagents

Reagents were as follows: BK, 40 kDa FITC-Dextran, hyaluronidase, arachidonic acid (AA) anti-Mouse IgG FITC, anti-Rabbit IgG TRICT, anti-B2 receptor, and anti-β-actin (Sigma Chemical); anti-COX-2 and anti-mPGES-1 (Cayman Chemicals); IKK inhibitor VII, anti-p65 and anti CD31 (Millipore); anti-ZO-1 (BD Transduction); anti-p-β catenin (Ser33, 37/Thr41) and anti-β catenin (Cell Signaling); anti VE-Cadherin (e-Bioscience); anti-H2A (Santa Cruz), anti-phospho-p65 (Bioss); anti-CD133 (Boster Immunoleader). Fasitibant ((4*S*)-4-amino-5-{4-[4-(2,4-dichloro-3-{[(2,4-dimethylquinolin-8-yl) oxy]methyl}benzenesulfonamido)oxane-4-carbonyl]piperazin-1-yl}-*N,N,N*-trimethyl-5-oxopentan-1-aminium chloride dihydrochloride or MEN16132) (batch number 2010/02), was synthesized in Menarini Ricerche, (Chemistry Development Department, Pisa, Italy).

### Cell Culture

Human Umbilical cord vein endothelial cells (HUVEC) were from Cambrex and were maintained in basal EGM-2 and 10% FBS (Hyclone). Cells were split 1∶3 twice a week, and used until they reached passage 7. Primary human hematopoietic-proangiogenic cells (PACs, CD133+ and CD34+ cells) isolated from human bone marrow, were from Lonza (2M-102), and were maintained in IMDM and 15% FBS. Cells were split 1∶2 once a week, and used until they reached passage 5.

### RT-PCR

Bradykinin 1 and 2 receptors mRNA expression were measured through differential RT-PCR. Cells were maintained for 24 hrs in 0.1%, 10% FBS or treated with BK (1 µM) and total RNA was obtained using RNA mini kit (Qiagen). RNA (0.5 µg) was reverse transcribed using a RT-PCR kit (Applied Biosystems). Differential RT-PCR was carried out by using the following primers [37]: B2R (339 bp) 5′-GTCCATGGGCCGGATGCGCGG-3′ (sense), 5′- CGATGCAGCGTATCCAGGAAGGTGC-3′ (antisense); B1R (437 bp) 5′-GGCAGAAATCTACCTGGCCAACC-3′ (sense), 5′GCCAGTGGTAGGAGGAAACCCAG-3′ (antisense). Results were normalized with GAPDH (196 bp) 5′-CCATGGAGAAGGCTGGGG-3′ (sense) and 5′-CAAAGTTGTCATGGATGACC-3′ (antisense).

### Western Blot

3×10^5^ cells (HUVEC) or 1.5×10^5^ cells (PACs) were plated in 6 or 3 cm diameter dishes, respectively. After 24 h, cells were exposed to 0.1% FBS or BK in presence/absence of fasitibant or IKK inhibitor VII. Cells were scraped in a lysis buffer containing 50 mM Tris HCl (pH 7.4), 150 mM NaCl, 1 mM EGTA, 10 mM NaF, 1% Triton and 1% protease inhibitor cocktail. To prepare the nuclear fractions, 8×10^5^ cells were plated in 10 cm diameter dishes. After 24 h, cells were exposed to 0.1% FBS (Control condition) or BK in presence/absence of fasitibant. Cells were then suspended in extraction buffer containing (in mM) 10 HEPES, 1 DTT, 10 KCl, 50 NaF, 0.1 EDTA, 0.1 EGTA, 1 Na_3_VO_4_, 0.5 PMSF and 0.1 NP-40 at 4°C, homogenized, and centrifuged at 1,000 *g* for 10 min to separate the nuclei. The supernatant was centrifuged at 13,000 *g* for 15 min three times to yield the cytosolic fraction. The nuclear fraction was lysed in buffer containing (in mM) 20 HEPES, 1 EDTA, 1 EGTA and 0.5 PMSF and stored in −80°C before use.

Equal amounts (50 µg) of protein were separated by SDS-PAGE onto a gradient 4–12% gel and transferred to a nitrocellulose membrane. The membranes were blocked (1 h) in a solution of 5% (wt/vol) milk and then incubated overnight at 4°C with the primary antibodies: anti-B2 Receptor, anti- p-β catenin (Ser33, 37/Thr41), anti-COX-2, anti-phospho-p65 and anti-p65 (each 1∶1000) or anti mPGES-1 (1∶200) and normalized for β-Actin (1∶10,000), H2A (1∶1000, for nuclear fraction) or β catenin (1∶1000). After 1 hr incubation in a secondary antibody anti IgG HRP (diluted 1∶2500, Promega), the immunoreaction were revealed by chemioluminescence.

### Permeability

HUVEC were seeded at 1×10^5^ on collagen-coated insert membranes (Corning) with 0.4 µm diameter pores, and the inserts were placed in a 12 multiwell plate, for 48 h. Monolayers were treated with BK (0.1–1–10 µM) with/without fasitibant (0.1–1–10 µM), then a 40 kDa FITC-Dextran (10 µM), was added on top of cells, allowing the fluorescent molecules to pass through the cell monolayer. The extent of permeability was determined in a time range (0–45 min) by measuring the fluorescence in a plate reader (Tecan), at 485/535 nm, excitation/emission, respectively. Arbitrary values were plotted against time.

### Immunofluorescence

Cells were cultured on coverslips, treated with BK 1 or 10 µM in presence/absence of fasitibant and then fixed in paraformaldheyde 4% or cold acetone (5 min), washed in PBS and incubated with BSA 3% (45 min). Cells were then incubated for 16 hrs with anti- ZO-1 (1∶50) or anti-p65 (1∶40) or anti-VE-cadherin (1∶60) antibodies, in PBS containing 0.5% BSA. After incubation with the secondary antibody anti-Mouse IgG FITC (ZO-1) or anti-Rabbit IgG TRICT, (p65 and VE-cadherin) (1 h), cells were washed and the coverslips mounted with Mowioll 4–88 (Calbiochem) and images of cells were taken using a Nikon Eclipse TE 300 inverted microscope by a digital camera and NIS Element software (for ZO-1, VE-cadherin and p65, images at 20–60 X magnification) or using a confocal microscope Zeiss LSM700 (VE-cadherin, at 60 X magnification).

### BrDU Labeling Assay

Cell proliferation was determined by 5-bromo-2′-deoxy-uridine (BrdU) incorporation using a colorimetric ELISA according to the manufacturer’s instructions (Roche). Briefly, 1.5×10^3^ cells were seeded in 96 multiplate. After 24 hrs cells were incubated with BK (0.1–1–10 µM) in the presence/absence of fasitibant (0.1–1 µM) for 10 hrs. BrdU was added during the late stage (4 h) of incubation. Stained cells were counted with a light microscope (Nikon Eclipse E400) at 20 X magnification. Data are reported as BrdU labeled cell/well.

### Wound Assay

Endothelial cells were seeded into 24-well plates (1×10^5^ cells/well) and incubated for 24 h to grow into a confluent monolayer. Then, the monolayer was scraped using a sterile 20–200 µl micropipette tip to create a wound of ±1 mm width. The cells were washed twice with PBS and fresh medium containing test substances (BK: 0.1–1–10 µM, with/without fasitibant 1 µM) was added with ARA-C (2.5 µg/ml) to inhibit cell proliferation. Images of the wound in each well were acquired from 0 to 12 hrs under a phase contrast microscope at 20 X magnification. Results are expressed as area of migrated cells, 12 h vs. time 0. After 12 h, the cells were stained with Hoechst 33342, and images were obtained using as described above.

### *In vitro* Angiogenesis Model

HUVEC were pre-treated with IKK inhibitor VII (0.2 µM for 30 min) and then exposed to BK (1 µM) or treated with fasitibant (1 µM) in presence/absence of BK. Cells were then plated onto a thin layer (300 µl) of basement membrane matrix (Matrigel; BD Biosciences) in 24-well plates at 6×10^4^ cells/well in EBM. After 12 h, the medium was removed and the cells were fixed, and stained using DY554 phalloidin (Thermo Fisher Scientific) for F-actin. Images of cells were obtained as reported above. Quantification of tubular structures and photomicrographs were performed as previously described [38].

### Sprouting Formation *in vitro*

Cytodex microcarrier beads (500 mg) (Sigma) were swollen in 100 µl PBS (2 h) and then autoclaved (121°C, 20 min). 3×10^5^ HUVEC were seeded onto 1.000 cytodex microcarrier beads in 0.5 ml of EGM, 20% FBS. The mixture was incubated at 37°C to allow cell attachment and shaken every 30 min for 6 h. The HUVEC-coated beads were washed in serum free medium and added to 2.5 mg/ml fibrinogen solution. 15 µl of mixture were plated in 12 well plate in the presence of thrombin allowing the polymerization at room temperature. The gel was maintained with medium supplemented with 1% FBS. HUVEC, were treated with BK in presence/absence of fasitibant. Experiment was maintained for 4 days. Data are reported as quantification of total length of pseudocapillary like structures.

### *In vivo* Matrigel Angiogenesis Assay

This study was carried out in strict accordance with the recommendations in the Guide for the Care and Use of Laboratory Animals of the University of Siena (Polo Scientifico San Miniato). The protocol was approved by the Committee on the Ethics of Animal Experiments of the Azienda Ospedaliera Universitaria Senese (Permit Number: D-08032010). Further, all procedures were carried out in accordance with the Italian law (Legislative Decree no. 116, 27 January 1992), which acknowledges the European Directive 86/609/EEC. Injection of matrigel was performed without anesthesia, and all efforts were made to minimize suffering. Mice were sacrified with carbon dioxyde.

Similarly, for the Osteoarthritis assay (see below), all animal care and experimental procedures were conducted in compliance with the principles and guidelines of the European Union (2010/63/UE) and the Italian government regulations, and approved by the ethical committee of Menarini Ricerche.

C57 black mice (20–25 g) were kept in temperature- and humidity-controlled rooms (22°C, 50%) with lights on from 07∶00 to 19∶00, water and food available ad libitum. BK and fasitibant were diluted in Matrigel (Becton Dickinson, growth factors and phenol red-free) on ice to a final concentration of 1 µg/ml and 1 µM, respectively. C57/B6J mice (12 week old) were subcutaneously injected in the dorsal midline region with 0.5 ml of Matrigel alone or with Matrigel containing the stimuli. After 10 days mice were euthanized and implants harvested. Plugs were resuspended in 1 ml of Drabkin’s solution for 18 h at 4°C. Hemoglobin concentration was determined by absorbance at 540 nm and compared with a standard curve (Sigma).

### Induction of Osteoarthritis in Rats

Osteoarthritis was induced by intra-articular (i.ar.) injection of monosodium iodoacetate (MIA) solution in the knee joint [21], [22]. Male rats (Wistar, 250–280 g, Harlan laboratories) were divided in 4 groups (five animal for group), anesthetized with pentobarbital (40 mg/kg, 3 ml/kg i.p.) and treated with a single i.ar. injection of saline (control, 25 µl), MIA (1 mg/25 µl), fasitibant (10 µg/25), or MIA+fasitibant into the joint space of both knees. I.ar. injections were performed through the intrapatellar ligament after shaving the skin, and was followed by a gentle flexion of the knee. Solutions were prepared under sterile conditions and injected by using a 50-µl Hamilton microliter syringe with a 6-mm, 27-gauge needle that was inserted into the joint for approximately 2 to 3 mm. After three days, animals were anesthetized with urethane (1.2 g/kg, i.p.): synovial fluid was washed out from right knee and the synovial capsule obtained from the left knee. The synovial fluid washing was performed with 100 µl of saline, and the recovered volume centrifuged (5 min at 4°C at 10,000 g) and conserved at −80°C for VEGF and PGE-2 measurements. The synovial capsule was embedded in Tissue-Tek O.C.T. (Sakura, San Marcos, USA), for histology. The doses of MIA and fasitibant were selected on the basis of previous experiments [21], [22].

### Immunohistochemical Analysis

Six-µm-thick cryostat sections from tissue samples were processed for immunohistochemical and hematoxylin and eosin staining. For histopathological analysis of CD31 or CD133 we used hematoxylin and immunohistochemical staining. After inactivating of endogenous peroxidase activity and blocking of cross-reactivity with 3% BSA the sections were incubated at 4°C for 18 h with a diluted solution of CD31 (1∶120, Millipore) or CD133 (1∶70, Boster immunoleader). Location of the primary antibodies was achieved by subsequent application of a biotin-conjugated antiprimary antibody, a streptavidin-peroxidase and diaminobenzidine (Sigma). The staining was developed using a commercial immunoperoxidase staining kit following the manufacturer’s instruction (the biotin–streptavidin complex method, Millipore). The slides were counter-stained with hematoxylin. Negative controls were established by replacing the primary antibody with PBS. Specific staining for CD31 or CD133 was categorized as either positive or negative based on the presence of brown-color staining. Images were analyzed using Nikon Eclypse T200 (20x magnification). Quantification of CD31 was performed counting 10 random field/section for slides.

### Immuno-assays for VEGF

Quantification of rat VEGF in the synovial fluid was determined by ELISA using a Rat Quantikine kit (R&D System). Synovial fluid diluted (1∶5) in the standard diluents, was treated with hyaluronidase (600 U) for 1 h at 37°C on a shaker before measurements and assayed as indicated in manufacturer’s instructions. Results are expressed as pg/ml.

### PGE-2 EIA Kit

PGE-2 was measured by an EIA kit (ENZO lifetech). Cells were exposed to BK (3, 8 or 24 h) in presence/absence of fasitibant and treated with 10 µM arachidonic acid (AA). Cell culture supernatants were assayed at a final dilution of 1∶10. PACs were exposed to BK (8 h) in presence/absence of fasitibant and treated with 10 µM arachidonic acid (AA); cell culture supernatant was assayed as indicated in manufacturer’s instructions. PGE-2 was expressed as [pg^/^ml], normalized to total protein concentration.

### Data Analysis and Statistical Procedures

Results are either representative or average of at least three independent experiments done in triplicate. Statistical analysis was performed using ANOVA test, Bonferroni test and t test for unpaired data (Prism, GraphPad). P<0.05 was considered statistically significant.

## Supporting Information

Figure S1 BK stimulation does not change BR1 or BR2 mRNA expression. (A) mRNA expression for B1 and B2 receptors in HUVEC treated with 0.1% FBS (first lane), 10% FBS (second lane) or BK (1 µM, third lane). The ratio between B2R over GAPDH is reported.
(PDF)

Figure S2 BK-induced changes of endothelial junctions signals and β-catenin phosphorylation are blocked by fasitibant in HUVEC. (A) Graphs represent the percentage of positive cells for VEC (leftt graph) or ZO-1 (right graph) immunofluorescence. (B) Graphs represent the optical densities related to the ratio between phospho-β catenin over β catenin. A.D.U. (arbitrary density unit), **p<0.01 and ***p<0.001 versus control, ###p<0.001 versus BK. Numbers represent mean ± SD of three experiments.
(PDF)

Figure S3 Fasitibant impairs endothelial cell growth and migration. (A) Cells were exposed to BK (1 µM), or to BK in presence/absence of fasitibant (0.1–1 µM) for 10 hrs and growth was evaluated by BrdU incorporation. Data are reported as cell number counted/well. Numbers represent mean ± SEM of three experiments run in triplicate. (B) Scratch wound healing assay on HUVEC treated with 0.1% FBS (a), BK (1 µM) (b), fasitibant (1 µM) (c), fasitibant+BK (d). (C) Quantification of cell migration was reported as area of migrated cells. ***p<0.001, compared to untreated cells; ###p<0.001 to BK-treated cells. Numbers represent mean ± SEM of three experiments run in triplicate.
(PDF)

Figure S4 BK-induced angiogenesis *in vitro* is reduced by B2R blockade. (A) Representative pictures of microcarrier-HUVEC in 0.1% FBS (a), exposed to BK (1 µM) (b), to fasitibant (1 µM) (c), to fasitibant+BK (d). (B) Quantification of sprouting lenght (µm); Numbers represent mean ± SEM of three experiments run in triplicate. ***p<0.001, compared to untreated cells; ###p<0.001 to BK-treated cells. (C) Representative pictures of capillary structures (diameter) in microcarrier-HUVEC treated with BK (1 µM, panel a) in presence/absence of fasitibant (1 µM, panel b). (D) Quantificationof sprouting diameter (µm); Numbers represent mean ± SEM of three experiments run in triplicate. ***p<0.001, compared to untreated cells; ###p<0.001 to BK-treated cells.
(PDF)

Figure S5 BK stimulates translocation/activation of NF-κB in HUVEC. (A–B) Graphs represent the optical densities related to the ratio between nuclear p65 over H2A, or cytoplasmic p65 over actin. A.D.U. (arbitrary density unit), numbers represent mean ± SD of three experiments. (A) Comparison between: cytoplasmic vs. nuclear fraction at time: 0 p<0.001, 30 min p<0.05, 60 min p<0.001; BK treatment vs. ctr in nuclear or cytoplasmic fraction at time:5 min p<0.001, 15 min p<0.001 and 30 min p<0.001. (B) Comparison between: cytoplasmic vs. nuclear fraction in control (0.1% FBS) condition p<0.001; BK treatment vs. ctr in nuclear or cytoplasmic fraction p<0.001, co-treatment between fasitibant and BK vs. BK p<0.001.
(PDF)

Figure S6 B2R expression in human circulating proangiogenic cells. Western blot analysis of B2 receptor in human circulating proangiogenic cells treated with 0.1% FBS (Ctr) or BK (1 µM) for 24 h. (Experiments are run three time; n = 3). The ratio between B2R over actin is reported.
(PDF)
