# Supplementary material for: Antagonism of Bradykinin B2 Receptor Prevents Inflammatory Responses in Human Endothelial Cells by Quenching the NF-kB Pathway Activation
Source: PLoS One. 2014 Jan 2;9(1):e84358. doi: 10.1371/journal.pone.0084358 (PMC3879294; doi:10.1371/journal.pone.0084358)
Supplement: Figure S1 — BK stimulation does not change BR1 or BR2 mRNA expression. (A) mRNA expression for B1 and B2 receptors in HUVEC treated with 0.1% FBS (first lane), 10% FBS (second lane) or BK (1 µM, third lane). The ratio between B2R over GAPDH is reported. (PDF) [file pone.0084358.s001.pdf]

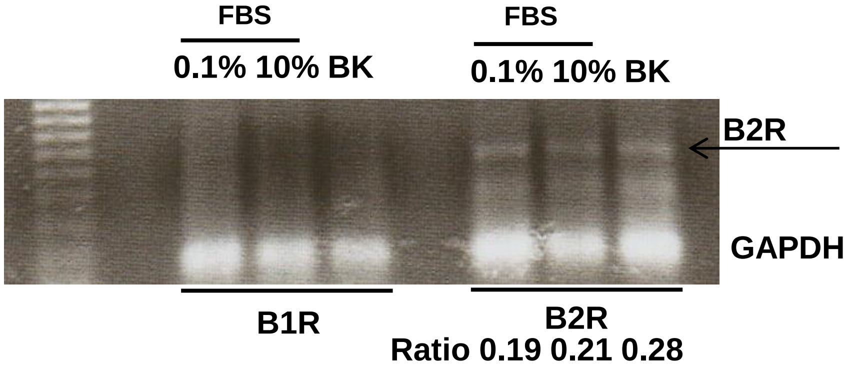

Figure S1 BK stimulation does not change BR1 or BR2 mRNA expression. (A) mRNA expression for B1 and B2 receptors in HUVEC treated with 0.1% FBS (first lane), 10% FBS (second lane) or BK (1 µM, third lane). The ratio between B2R over GAPDH is reported.
