# Supplementary material for: Antagonism of Bradykinin B2 Receptor Prevents Inflammatory Responses in Human Endothelial Cells by Quenching the NF-kB Pathway Activation
Source: PLoS One. 2014 Jan 2;9(1):e84358. doi: 10.1371/journal.pone.0084358 (PMC3879294; doi:10.1371/journal.pone.0084358)
Supplement: Figure S2 — BK-induced changes of endothelial junctions signals and β-catenin phosphorylation are blocked by fasitibant in HUVEC. (A) Graphs represent the percentage of positive cells for VEC (leftt graph) or ZO-1 (right graph) immunofluorescence. (B) Graphs represent the optical densities related to the ratio between phospho-β catenin over β catenin. A.D.U. (arbitrary density unit), **p<0.01 and ***p<0.001 versus control, ###p<0.001 versus BK. Numbers represent mean ± SD of three experiments. (PDF) [file pone.0084358.s002.pdf]

A

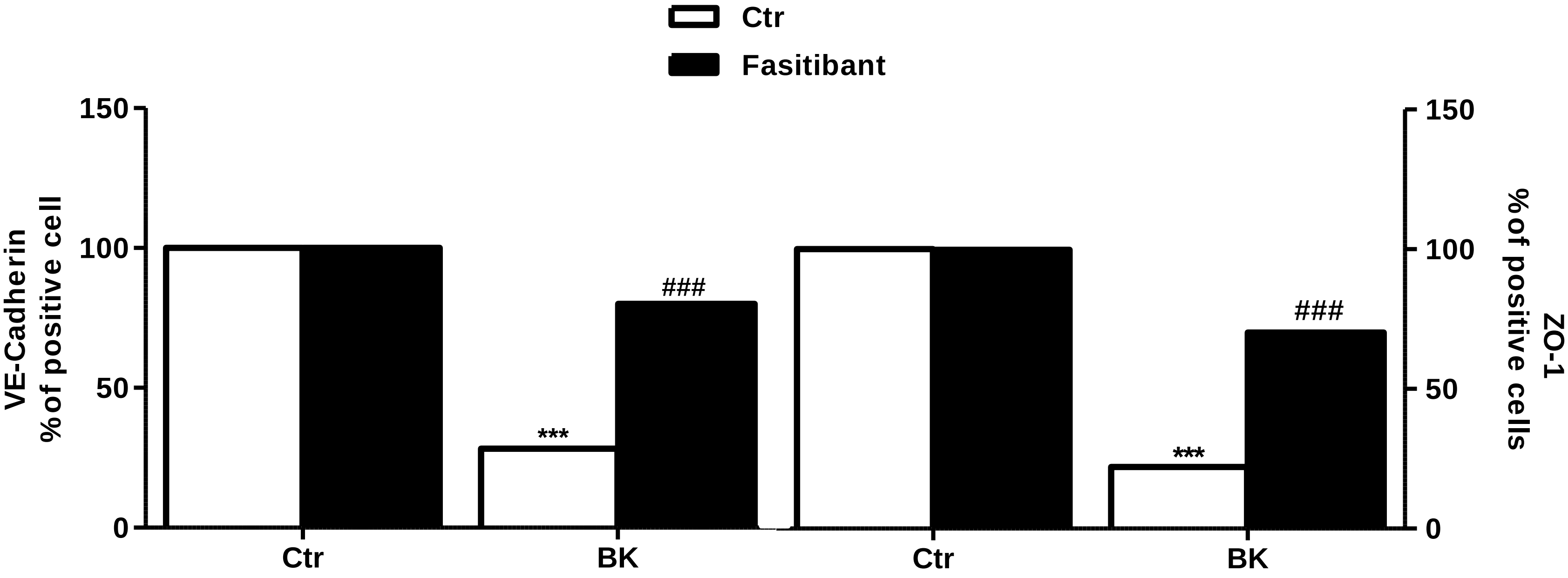

B

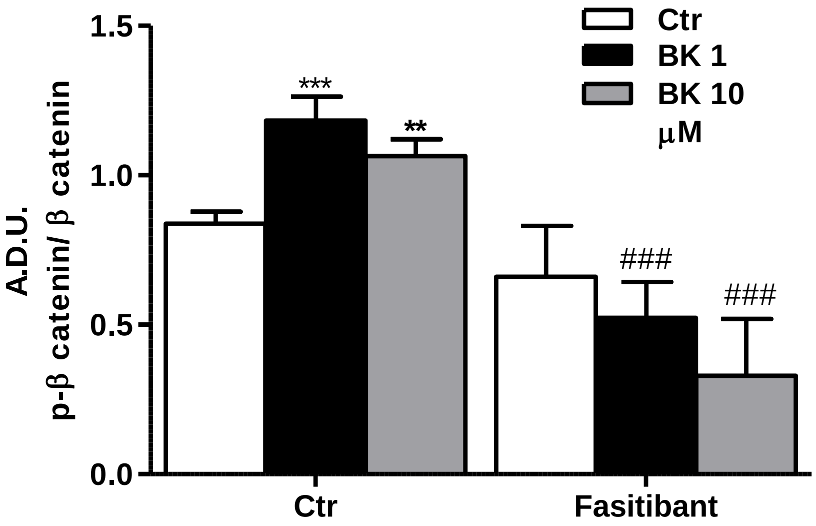

Figure S2 BK-induced changes of endothelial junctions signals and β-catenin phosphorylation are blocked by fasitibant in HUVEC. (A) Graphs represent the percentage of positive cells for VEC (leftt graph) or ZO-1 (right graph) immunofluorescence. (B) Graphs represent the optical densities related to the ratio between phospho-β catenin over β catenin. A.D.U. (arbitrary density unit), ** p <0.01 and *** p <0.001 versus control, ### p<0.001 versus BK. Numbers represent mean ± SD of three experiments.
