# Supplementary material for: Antagonism of Bradykinin B2 Receptor Prevents Inflammatory Responses in Human Endothelial Cells by Quenching the NF-kB Pathway Activation
Source: PLoS One. 2014 Jan 2;9(1):e84358. doi: 10.1371/journal.pone.0084358 (PMC3879294; doi:10.1371/journal.pone.0084358)
Supplement: Figure S3 — Fasitibant impairs endothelial cell growth and migration. (A) Cells were exposed to BK (1 µM), or to BK in presence/absence of fasitibant (0.1–1 µM) for 10 hrs and growth was evaluated by BrdU incorporation. Data are reported as cell number counted/well. Numbers represent mean ± SEM of three experiments run in triplicate. (B) Scratch wound healing assay on HUVEC treated with 0.1% FBS (a), BK (1 µM) (b), fasitibant (1 µM) (c), fasitibant+BK (d). (C) Quantification of cell migration was reported as area of migrated cells. ***p<0.001, compared to untreated cells; ###p<0.001 to BK-treated cells. Numbers represent mean ± SEM of three experiments run in triplicate. (PDF) [file pone.0084358.s003.pdf]

Figure S3

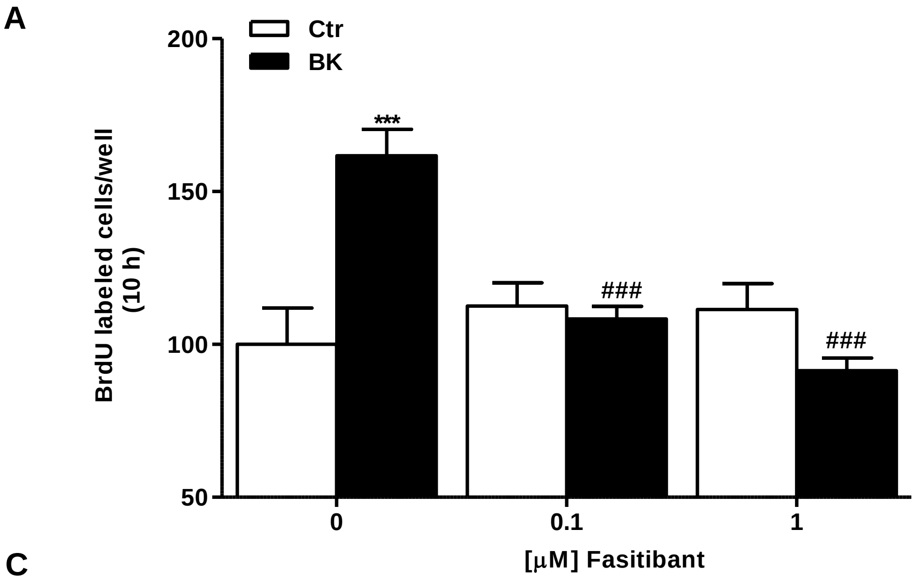

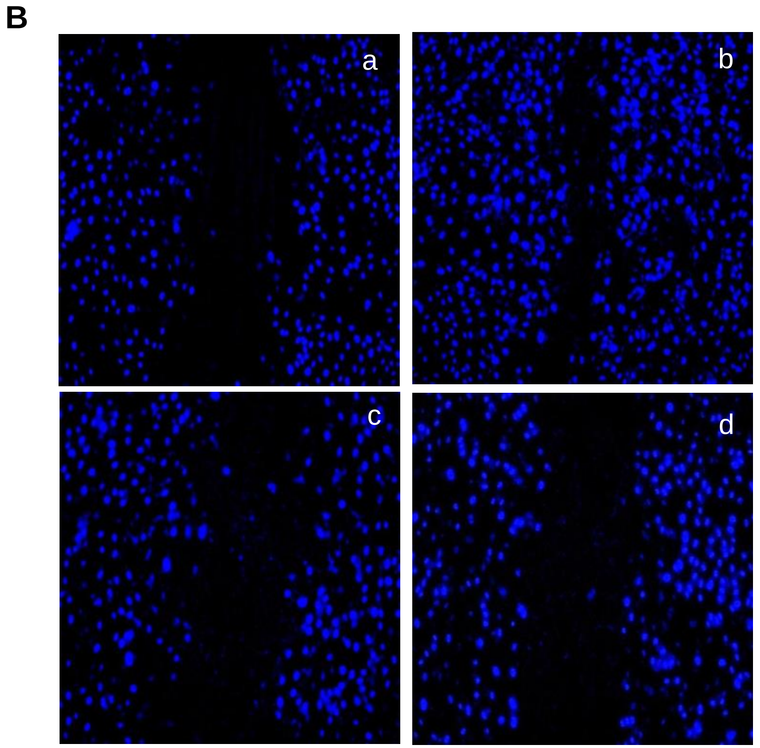

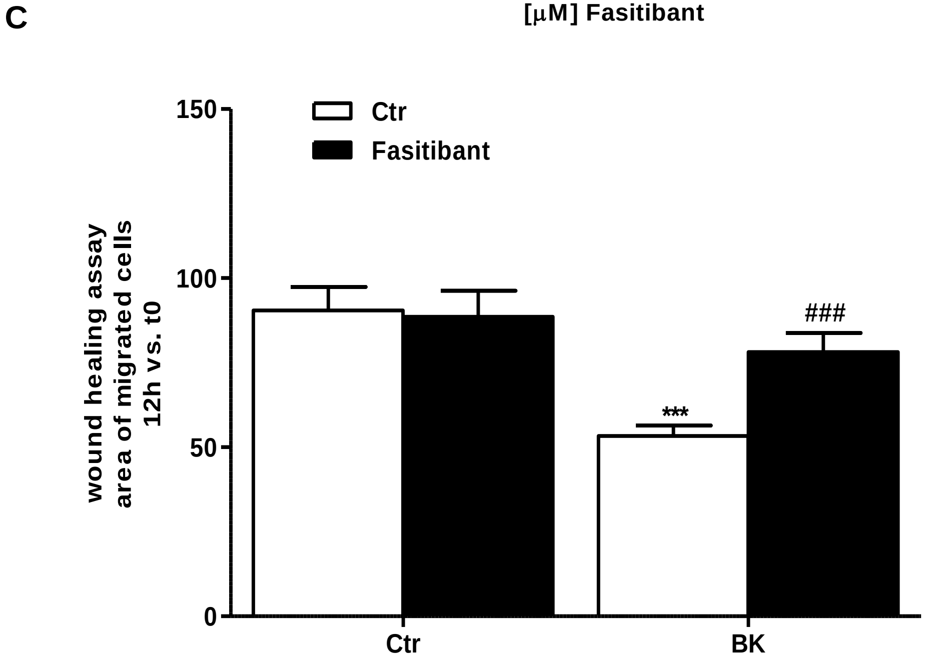

Figure S3 Fasitibant impairs endothelial cell growth and migration. (A) Cells were exposed to BK (1 μM), or to BK in presence/absence of fasitibant (0.1-1 μM) for 10 hrs and growth was evaluated by BrdU incorporation. Data are reported as cell number counted/well. Numbers represent mean ± SEM of three experiments run in triplicate. (B) Scratch wound healing assay on HUVEC treated with 0.1% FBS (a), BK (1 μM) (b), fasitibant (1 μM) (c), fasitibant + BK (d). (C) Quantification of cell migration was reported as area of migrated cells. *** $p < 0.001$, compared to untreated cells; ### $p < 0.001$ to BK-treated cells. Numbers represent mean ± SEM of three experiments run in triplicate.
