# Supplementary material for: Antagonism of Bradykinin B2 Receptor Prevents Inflammatory Responses in Human Endothelial Cells by Quenching the NF-kB Pathway Activation
Source: PLoS One. 2014 Jan 2;9(1):e84358. doi: 10.1371/journal.pone.0084358 (PMC3879294; doi:10.1371/journal.pone.0084358)
Supplement: Figure S4 — BK-induced angiogenesis in vitro is reduced by B2R blockade. (A) Representative pictures of microcarrier-HUVEC in 0.1% FBS (a), exposed to BK (1 µM) (b), to fasitibant (1 µM) (c), to fasitibant+BK (d). (B) Quantification of sprouting lenght (µm); Numbers represent mean ± SEM of three experiments run in triplicate. ***p<0.001, compared to untreated cells; ###p<0.001 to BK-treated cells. (C) Representative pictures of capillary structures (diameter) in microcarrier-HUVEC treated with BK (1 µM, panel a) in presence/absence of fasitibant (1 µM, panel b). (D) Quantificationof sprouting diameter (µm); Numbers represent mean ± SEM of three experiments run in triplicate. ***p<0.001, compared to untreated cells; ###p<0.001 to BK-treated cells. (PDF) [file pone.0084358.s004.pdf]

Figure S4

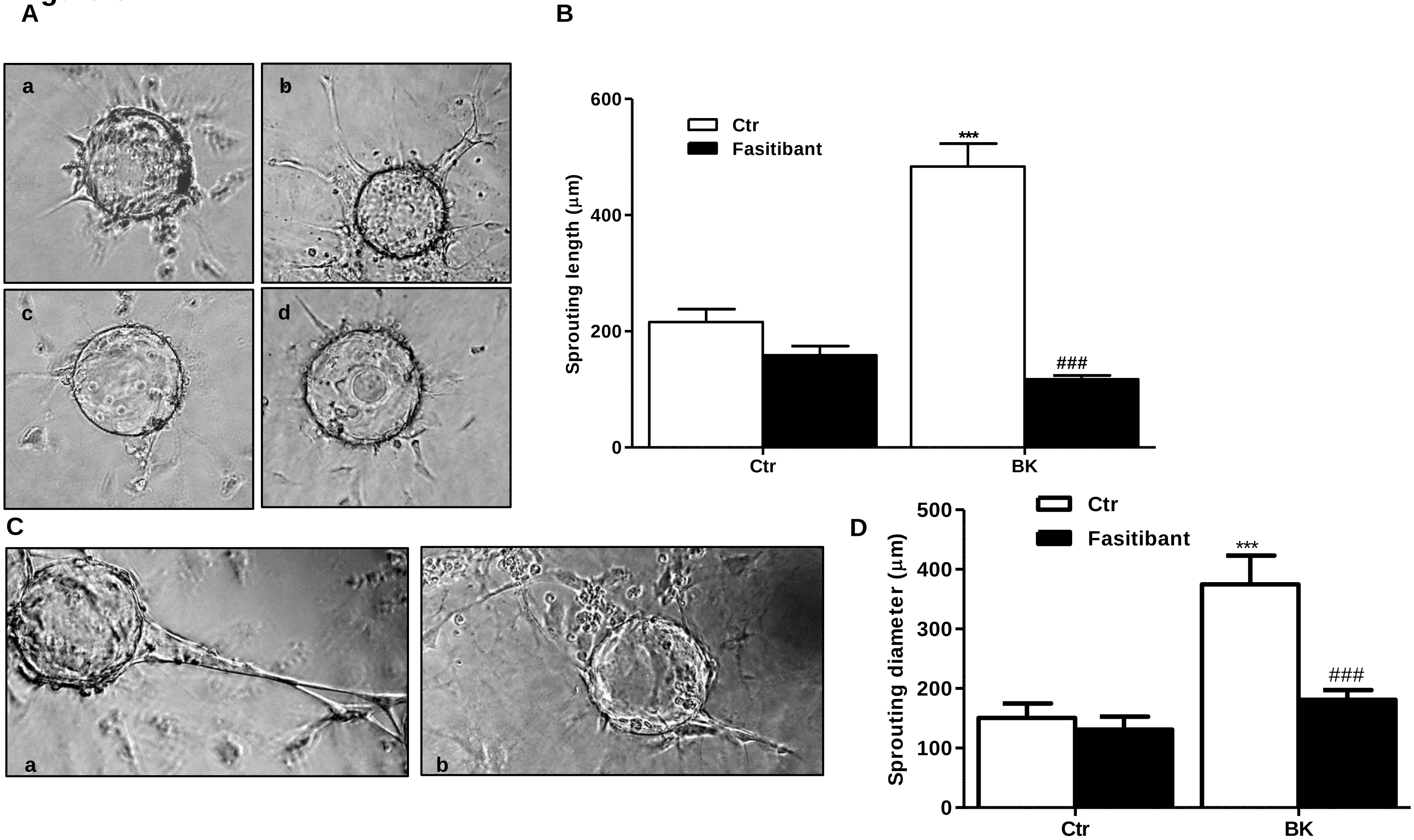

Figure S4 BK-induced angiogenesis *in vitro* is reduced by B2R blockade. (A) Representative pictures of microcarrier-HUVEC in 0.1% FBS (a), exposed to BK (1 μM) (b), to fasitibant (1 μM) (c), to fasitibant + BK (d). (B) Quantification of sprouting lenght (μm); Numbers represent mean ± SEM of three experiments run in triplicate. *** p< 0.001, compared to untreated cells; ### p< 0.001 to BK-treated cells. (C) Representative pictures of capillary structures (diameter) in microcarrier-HUVEC treated with BK (1 μM, panel a) in presence/absence of fasitibant (1 μM, panel b). (D) Quantification of sprouting diameter (μm); Numbers represent mean ± SEM of three experiments run in triplicate. *** p< 0.001, compared to untreated cells; ### p< 0.001 to BK-treated cells.
