# Supplementary material for: Antagonism of Bradykinin B2 Receptor Prevents Inflammatory Responses in Human Endothelial Cells by Quenching the NF-kB Pathway Activation
Source: PLoS One. 2014 Jan 2;9(1):e84358. doi: 10.1371/journal.pone.0084358 (PMC3879294; doi:10.1371/journal.pone.0084358)
Supplement: Figure S5 — BK stimulates translocation/activation of NF-κB in HUVEC. (A–B) Graphs represent the optical densities related to the ratio between nuclear p65 over H2A, or cytoplasmic p65 over actin. A.D.U. (arbitrary density unit), numbers represent mean ± SD of three experiments. (A) Comparison between: cytoplasmic vs. nuclear fraction at time: 0 p<0.001, 30 min p<0.05, 60 min p<0.001; BK treatment vs. ctr in nuclear or cytoplasmic fraction at time:5 min p<0.001, 15 min p<0.001 and 30 min p<0.001. (B) Comparison between: cytoplasmic vs. nuclear fraction in control (0.1% FBS) condition p<0.001; BK treatment vs. ctr in nuclear or cytoplasmic fraction p<0.001, co-treatment between fasitibant and BK vs. BK p<0.001. (PDF) [file pone.0084358.s005.pdf]

Figure S5

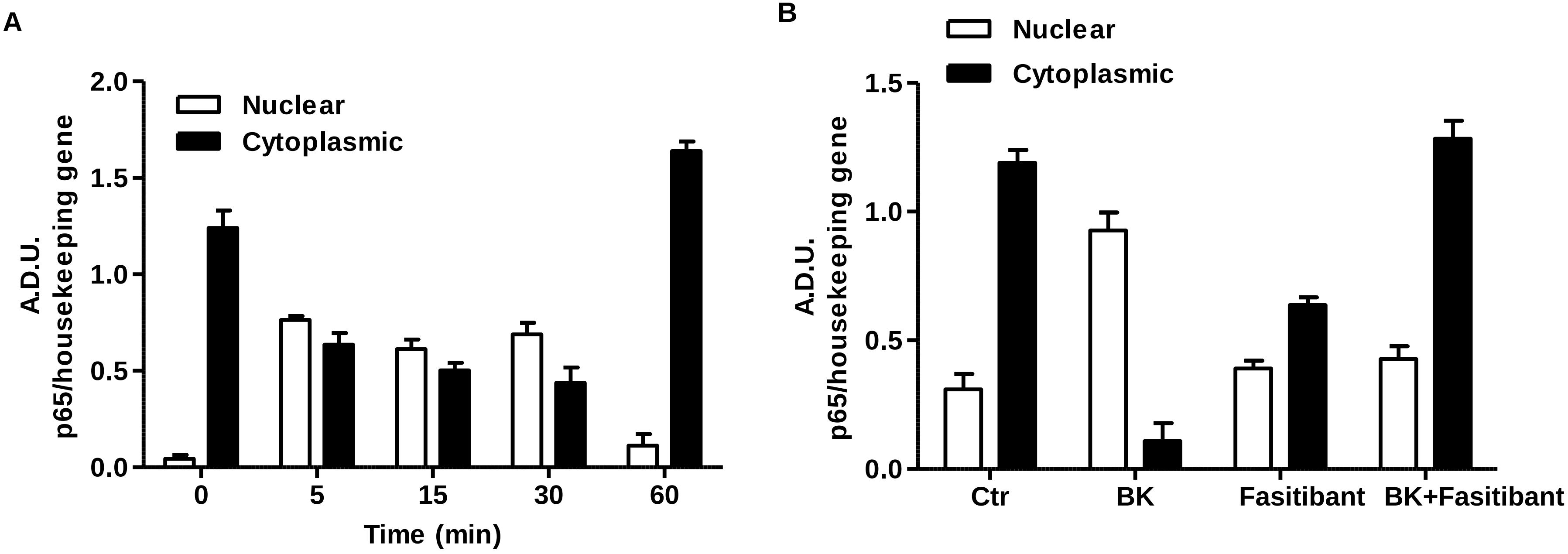

Figure S5 BK stimulates translocation/activation of NF-κB in HUVEC. (A-B) Graphs represent the optical densities related to the ratio between nuclear p65 over H2A, or cytoplasmic p65 over actin. A.D.U. (arbitrary density unit), numbers represent mean ± SD of three experiments. (A) Comparison between: cytoplasmic vs. nuclear fraction at time: 0 $p<0.001$, 30 min $p<0.05$, 60 min $p<0.001$; BK treatment vs. ctr in nuclear or cytoplasmic fraction at time:5 min $p<0.001$, 15 min $p<0.001$ and 30 min $p<0.001$. (B) Comparison between: cytoplasmic vs. nuclear fraction in control (0.1% FBS) condition $p<0.001$; BK treatment vs. ctr in nuclear or cytoplasmic fraction $p<0.001$, co-treatment between fasitibant and BK vs. BK $p<0.001$.
