# Supplementary material for: Antagonism of Bradykinin B2 Receptor Prevents Inflammatory Responses in Human Endothelial Cells by Quenching the NF-kB Pathway Activation
Source: PLoS One. 2014 Jan 2;9(1):e84358. doi: 10.1371/journal.pone.0084358 (PMC3879294; doi:10.1371/journal.pone.0084358)
Supplement: Figure S6 — B2R expression in human circulating proangiogenic cells. Western blot analysis of B2 receptor in human circulating proangiogenic cells treated with 0.1% FBS (Ctr) or BK (1 µM) for 24 h. (Experiments are run three time; n = 3). The ratio between B2R over actin is reported. (PDF) [file pone.0084358.s006.pdf]

Figure S6

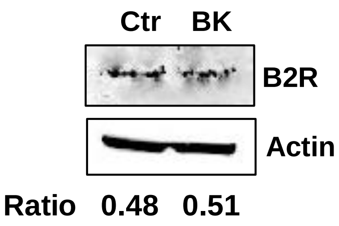

Figure S6 B2R expression in human circulating proangiogenic cells. Western blot analysis of B2 receptor in human circulating proangiogenic cells treated with 0.1% FBS (Ctr) or BK (1 μM) for 24 h. (Experiments are run three time; n=3). The ratio between B2R over actin is reported.
